# Physical activity self-efficacy online intervention for adults with obesity: protocol for a feasibility study

**DOI:** 10.1186/s40814-024-01468-6

**Published:** 2024-02-26

**Authors:** Seungmin Lee, Kevin Lahoda, Nicholas D. Myers, Andrew Horowitz, Kenneth Chiu, Lina Begdache, Eldad Einav

**Affiliations:** 1 Division of Health and Wellness Studies, Binghamton University, Binghamton, USA; 2 Department of Art, Architecture and Design, Lehigh University, Bethlehem, USA; 3 Department of Kinesiology, Michigan State University, East Lansing, USA; 4 Department of Theatre, Binghamton University, Binghamton, USA; 5 Department of Computer Science, Binghamton University, Binghamton, USA; 6 Guthrie Lourdes Hospital, Binghamton, USA

**Keywords:** Exercise, eHealth, Internet, Information and communication technology

## Abstract

**Background:**

Even without weight loss, adults with obesity can greatly benefit from regular physical activity. The Physical Activity Self-efficacy (PAS) intervention is an online behavioral intervention newly developed to promote physical activity in adults with obesity by providing capability-enhancing learning opportunities. The objective of this manuscript is to describe the protocol for a feasibility study designed to investigate the feasibility and acceptability of implementing the PAS online intervention for adults with obesity recruited from a local weight management center in the United States of America (USA).

**Methods:**

The study design is a prospective, double-blind, parallel-group individual randomized pilot trial. Thirty participants will be randomly assigned to the PAS group or usual care group to achieve a 1:1 group assignment. Recruitment of participants is scheduled to begin on 1 March 2024 at a local weight management center within a private healthcare system in the USA. There are six eligibility criteria for participation in this study (e.g., a body mass index ≥ 25.00 kg/m^2^). Eligibility verification and data collection will be conducted online. Three waves of data collection will take up to 14 weeks depending on participants’ progress in the study. The primary feasibility outcomes in the study will be: (a) participation rate, (b) engagement behavior, and (c) a preliminary effect size estimate for the effect of the PAS intervention on physical activity. Instruments designed to measure demographic information, anthropometric characteristics, self-efficacy, and acceptability will be included in the survey battery. A research-grade accelerometer will be used to measure free-living physical activity objectively. Data will be analyzed using descriptive statistics and inferential statistical models under an intention-to-treat approach.

**Discussion:**

Results are intended to inform the preparation of a future definitive randomized controlled trial.

**Trial registration:**

ClinicalTrials.gov, NCT05935111, registered 7 July 2023.

**Supplementary Information:**

The online version contains supplementary material available at 10.1186/s40814-024-01468-6.

## Background

The relationship between physical activity (PA) and health is not well understood by adults with obesity, not even by some health professionals. There is a misguided understanding that PA in adults with obesity should result in weight loss to obtain health benefits [1, 2]. However, there is strong evidence that, even without weight loss, adults with obesity can greatly benefit from regular PA for multiple reasons (e.g., relative reduction in incidence or progression of a chronic disease; improvements in insulin sensitivity, blood pressure, and body composition; attenuated weight gain; etc.) [3, 4]. In addition, previous meta-analyses have reported that promoting physical activity may help improve the mental health of adults with obesity [5, 6]. Having PA mainly for weight loss is problematic because adults with obesity may feel disheartened and lose motivation for PA if weight loss is not achieved at a satisfactory rate, or not at all. As expected, there is persistent evidence that most adults with obesity do not meet public health guidelines for PA [7, 8], for example, 150 min per week of moderate PA [3, 9, 10].

Based on extensive literature review [2, 11–26], we propose to develop and test a new online behavioral intervention, named the Physical Activity Self-efficacy (PAS) intervention. The PAS intervention is an online behavioral intervention specifically designed to promote PA in adults with obesity by providing capability-enhancing learning opportunities. The PAS intervention is: (a) population-tailored (i.e., adults with obesity), (b) aimed to promote multi-dimensional PA (i.e., work-, transport-, domestic-, leisure-related PA), (c) theory-based (i.e., self-efficacy theory), (d) scalable and sustainable (i.e., online delivery), (e) implemented with device-based PA assessments (i.e., an accelerometer), and (f) collaborated with an external community partner (i.e., a local weight management center), to promote PA in adults with obesity. A recent systematic review showed that the majority of research- or commercial-grade online interventions did not address some unique barriers to PA from adults with obesity but only added typical PA advice into diet-focused interventions [27]. In the absence of the proposed intervention, promoting PA in adults with obesity will likely remain difficult in reality.

**Fig. 1:**
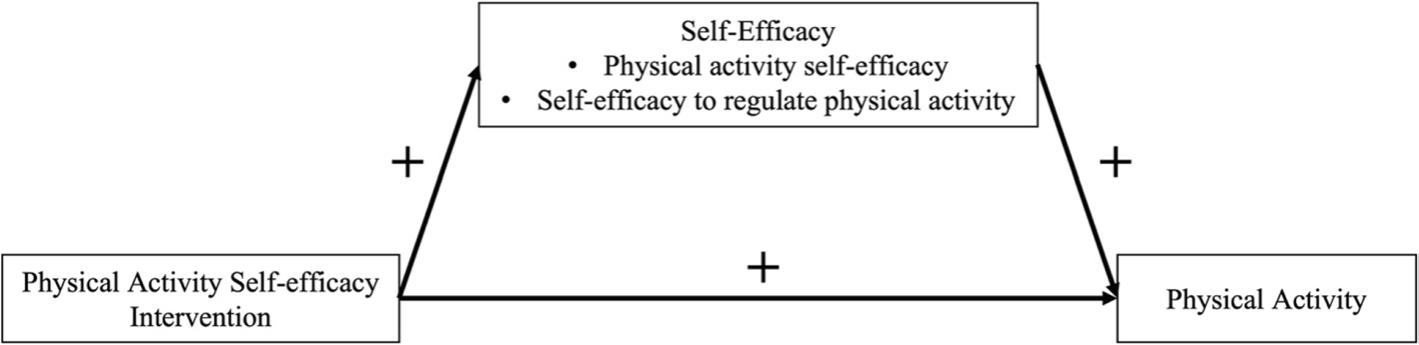
The conceptual model of the physical activity self-efficacy intervention

The PAS intervention builds off the Fun For Wellness (FFW) intervention [28–31]. Compared to FFW, the PAS intervention is more tailored for adults with obesity (i.e., at-risk subgroup of adults) and specifically designed to promote PA (i.e., not well-being) to achieve greater behavior change for their PA. Self-efficacy is specified as a mediating psychological variable in the conceptual model for the promotion of PA (see Fig. 1). Self-efficacy refers to domain-specific beliefs about their ability to execute differing levels of performance given situational demands [32–34]. There is a rich literature on the importance of targeting self-efficacy as a modifiable mediating variable in PA interventions [35–37]. The PAS intervention consists of 6 intervention components. Across the intervention components, there is a total of 30 introductory or post-introductory challenges in the PAS intervention. Precise reporting of the PAS intervention (e.g., how PAS builds off FFW) is provided in Appendix A, consistent with relevant recommendations [38, 39].

The objective of this manuscript is to describe the protocol for a feasibility study designed to investigate the feasibility and acceptability of implementing the PAS online intervention for adults with obesity recruited from a local weight management center in the United States of America (USA). Three specific aims will be investigated. 

### Aim 1

To determine the feasibility of implementing the PAS intervention (e.g., participation rate) for adults with obesity.

### Aim 2

To determine the acceptability of implementing the PAS intervention (e.g., engagement behavior) for adults with obesity.

### Aim 3

To provide a preliminary effect size estimate for each direct effect depicted in the conceptual model (see Fig. 1) for the PAS intervention (e.g., PAS  →  PA).

## Methods

This protocol was written based on the Standard Protocol Items: Recommendations for Interventional Trials (SPIRIT) [40, 41]. A SPIRIT flow diagram is provided in Table 1. A populated SPIRIT checklist is provided in Appendix B. The trial was registered at ClinicalTrials.gov, identifier: NCT05935111, registered 7 July 2023.

**Table 1:**
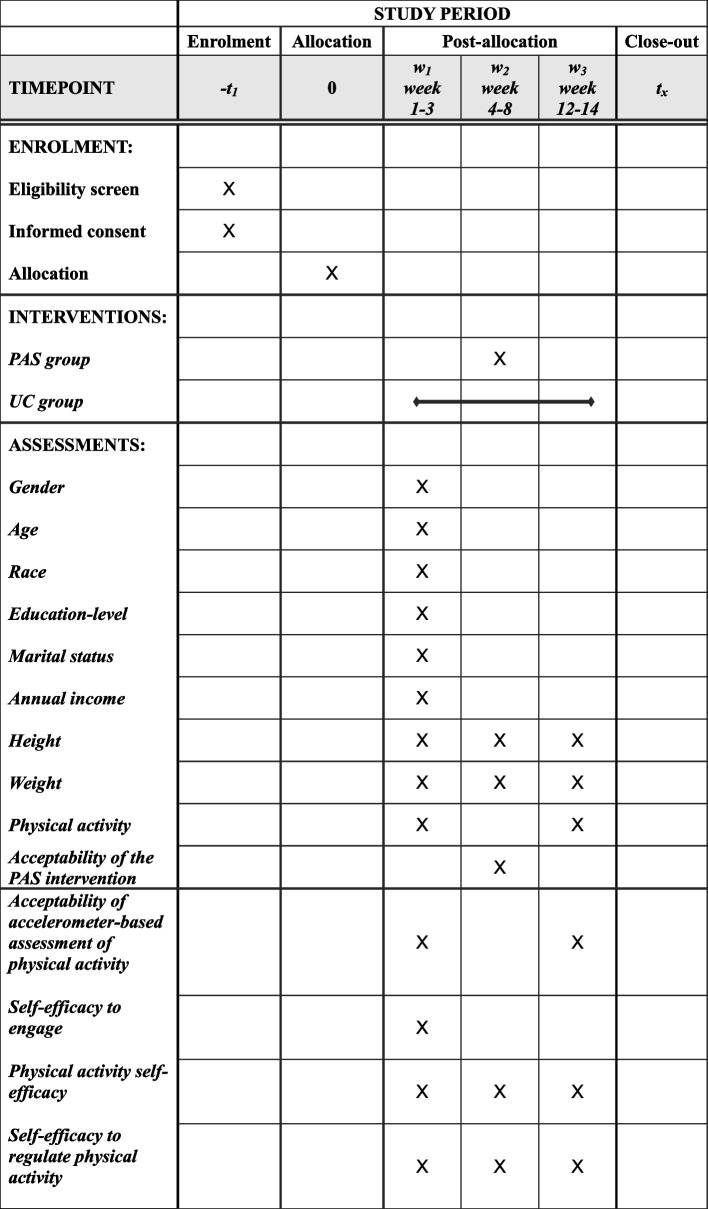
The SPIRIT flow diagram for the physical activity self-efficacy intervention feasibility study

*PAS* Physical Activity Self-efficacy, *UC* Usual Care

### Ethical approval

All procedures in this study involving human participants will be in accordance with the ethical standards of the institutional and national research committee. The institutional review board at Ascension provided necessary permission to conduct this study on 14 July 2023, RNY20230009. The procedures for confidentiality are provided in Appendix C.

### Study design

**Fig. 2:**
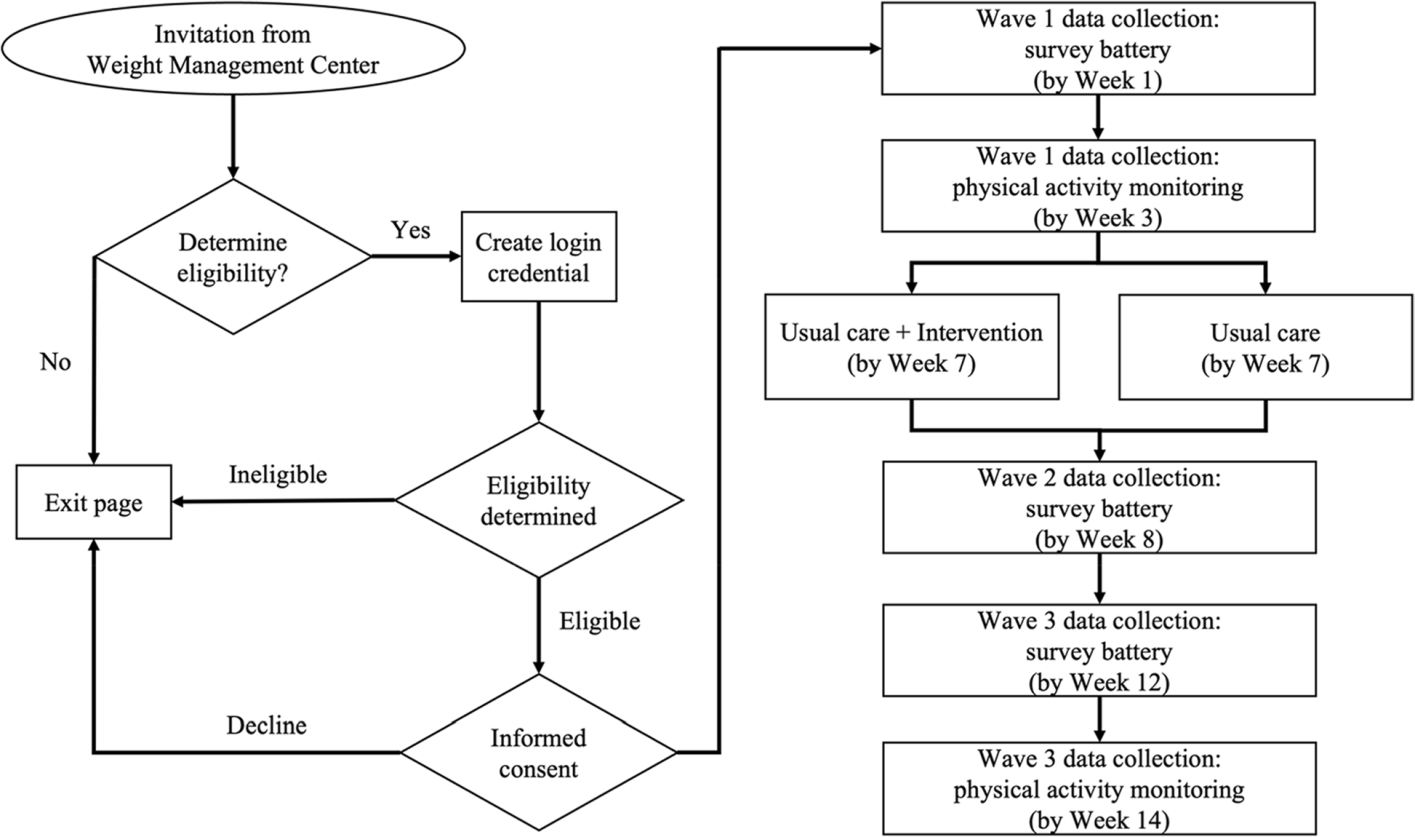
Flow chart for the implementation of the physical activity self-efficacy intervention

The study design is a prospective, double-blind, parallel-group individual randomized pilot trial. Recruitment of participants is scheduled to begin in March 2024 at a local weight management center within a private healthcare system in the USA. Eligibility verification and data collection will be conducted online. Three waves of data collection will take up to 14 weeks depending on participants’ progress in the study. Figure 2 provides a flow chart for recruitment of participants throughout data collection.

### Recruitment

Thirty participants will be recruited in this study. Patients who are enrolling in the weight management program provided by the center will be potential participants in the study (e.g., ~ 70 potential participants). They will be asked to consider their participation in this study by a research staff member who is affiliated with the center via in-person communication or remote communication. The research staff will provide only those interested in the study (e.g., ≤ 70 potential participants) with login information to the secure recruitment page on the PAS website which can then be accessed via their own device (e.g., smartphone). The PAS website will automatically stop recruitment once 30 participants provide their remote consent forms. We expect that the recruitment may take about one month.

### Eligibility and informed consent

There are six eligibility criteria that will be evaluated via self-report: (a) being between 18 and 64 years old, (b) a Body Mass Index (BMI) ≥ 25.00 kg/m^2^, (c) the ability to access the online intervention, (d) the absence of simultaneous enrollment in another intervention to promote PA (not counting the weight management program), (e) a willingness to comply with instructions for PA monitoring, and (f) a willingness to respond to study-related contacts. Detailed information and justifications for the criteria are provided in Appendix D. Those who meet all the eligibility criteria will be presented with the informed consent form to read and sign electronically.

### Sample size

The sample size (i.e., 30 participants) is based on the primary feasibility outcomes (see the feasibility outcome section for detailed information), the budgetary constraints, and the range of sample sizes observed in pilot and feasibility trials. Thirty participants may provide some data to use descriptive statistics for the primary feasibility outcomes (i.e., participation rate in Aim 1, engagement behavior in Aim 2). If the recruitment rate is 50% in this study, the targeted sample size can be used because the center usually has approximately 70 patients enrolled in their program. Budgetary constraints preclude enrollment of more than 30 participants in this study. Also, the targeted number of participants may provide some data about the preliminary effect size estimate for the direct effect of PAS to PA. The sample size in a feasibility study is not based on a sample size determination for a desired level of statistical power [42–44]. The target sample size in this study fits within the range of sample sizes often observed in pilot and feasibility trials [45].

### Randomization

Thirty participants will be randomly assigned to the PAS group or the Usual Care (UC) group to achieve a 1:1 group assignment. The randomization will be performed by the PAS intervention website (i.e., computer-generated random numbers). Neither participants nor the research staff will know which group a participant will be assigned into.

### UC group

The UC participants will proceed through the weight management program provided by the center. The login credentials for UC participants will provide access to a secure website to complete data collection at wave 1 (W1), wave 2 (W2), and wave 3 (W3). The UC participants will have the opportunity to receive up to $90 worth of Amazon electronic gift cards. Specifically, UC participants will receive: (a) $5 for completing the W1 survey battery, (b) $35 for completing the W1 PA monitoring for 7 days, (c) $5 for completing the W2 survey battery, (d) $5 for completing the W3 survey battery, and (e) $40 for completing the W3 PA monitoring for 7 days. The UC participants will be given 4 weeks of 24-h access to the PAS intervention immediately after data collection for the study is closed.

### PAS group

The PAS participants will proceed through the weight management program and will be given 4 weeks of 24-h access to the PAS intervention during data collection for this study. The login credentials for PAS participants will provide access to both the PAS intervention and to a secure website to complete data collection at W1, W2, and W3. The PAS participants will have the opportunity to receive up to $110 worth of Amazon electronic gift cards, which is the sum of the same gift card opportunities from the UC group (i.e., $90) and an additional $20 for completing at least 24 post-introductory challenges in the PAS intervention at W2.

### Study timeline

The W1 survey battery and the W1 PA monitoring will be included in data collection at W1. Participants will be asked to complete the W1 survey battery by week 1 (see the survey battery section for detailed information). Then, participants will be asked to complete the W1 PA monitoring by week 3 (see the PA monitoring section for detailed information). After W1, the 4-week access to the PAS intervention will be given to PAS participants by week 7. The W2 survey battery will be included in data collection at W2, but there will be no W2 PA monitoring based on the conceptual model of the PAS intervention. Participants will be asked to complete the W2 survey battery by week 8. The W3 survey battery and the W3 PA monitoring will be included in data collection at W3. Participants will be asked to complete the W3 survey battery by week 12. Then, participants will be asked to complete the W3 PA monitoring by week 14.

### Feasibility outcomes

The primary feasibility outcomes in the study will be: (a) participation rate (i.e., Aim 1), (b) engagement behavior (i.e., Aim 2), and (c) a preliminary effect size estimate for the direct effect of PAS to accelerometer-based assessment of PA (i.e., Aim 3). There will be secondary feasibility outcomes in Aim 1, 2, and 3. Threshold values for a traffic light system for each of the specific indicators that will be used to evaluate each aim are based on inferences drawn from the results of previous research [46, 47] and commonly used heuristics for Cohen’s *d* [48]. Data observed below a lower threshold (i.e., resembling red light) will suggest a potentially serious problem. Data observed above a lower threshold but below an upper threshold (i.e., resembling a yellow light) will suggest the need for caution. Data observed above an upper threshold (i.e., resembling green light) will suggest support for the feasibility of a future definitive Randomized Controlled Trial (RCT). The lower and upper bound threshold values for the traffic light system by aim are summarized in Table 2.

**Table 2:** Lower and upper bound threshold values for the traffic light system by aim

Aim	Lower Bound Threshold	Upper Bound Threshold
Aim 1		
Recruitment rate	< 40%	≥ 60%
Eligibility rate	< 60%	≥ 80%
Consent rate	< 80%	≥ 90%
Participation rates	< 50%	≥ 70%
Retention rates	< 40%	≥ 60%
Aim 2		
Engagement behavior in the PAS intervention	< 40%	≥ 60%
Quantitative acceptability of the PAS intervention	< 60%	≥ 80%
Dichotomous acceptability of the PAS intervention	< 60%	≥ 80%
Qualitative acceptability of the PAS intervention	at least one potentially serious problem	absence of a potentially serious problem
Quantitative acceptability of accelerometer-based assessment of physical activity	< 60%	≥ 80%
Dichotomous acceptability of accelerometer-based assessment of physical activity	< 60%	≥ 80%
Qualitative acceptability of accelerometer-based assessment of physical activity	at least one potentially serious problem	absence of a potentially serious problem
Aim 3		
Intention-to-treat approach	< 0.00	≥ 0.20

*PAS* Physical Activity Self-efficacy

### Aim 1

The Aim 1 is to determine the feasibility of implementing the PAS intervention for adults with obesity. The participation rate will be the primary feasibility outcome in Aim 1. There will be secondary feasibility outcomes (e.g., recruitment rate) in Aim 1.

#### Recruitment rate

Recruitment rate will be defined as the percentage of patients who select “yes” when asked if they are interested in determining if they are eligible for participation in this study: (*n*_interested_/[*n*_interested_ + *n*_not interested_]) × 100. The lower bound threshold is < 40%. The upper bound threshold is ≥ 60%.

#### Eligibility rate

Eligibility rate will be defined as the percentage of interested patients who are presented with the informed consent form: (*n*_eligible_/*n*_interested_) × 100. The lower bound threshold is < 60%. The upper bound threshold is ≥ 80%.

#### Consent rate

Consent rate will be defined as the percentage of eligible patients who consent to participate in this study: (*n*_consent_/*n*_eligible_) × 100. The lower bound threshold is < 80%. The upper bound threshold is ≥ 90%.

#### Participation rate

Participation rate at wave W, where W = W1 or W2 or W3, will be defined as the percentage of consented patients who provide usable data (e.g., non-missing data) at wave W: (*n*_usable data at wave W_/*n*_consent_) × 100. The lower bound threshold is < 50%. The upper bound threshold is ≥ 70%.

#### Retention rate

Retention rate through wave W, where W = W2 or W3, will be defined as the percentage of consented patients who provide usable data (e.g., non-missing data) at W1 through wave W: (*n*_usable data through wave W_/*n*_consent_) × 100. The lower bound threshold is < 40%. The upper bound threshold is ≥ 60%.

### Aim 2

The Aim 2 is to determine the acceptability of implementing the PAS intervention for adults with obesity. The engagement behavior in the PAS intervention will be the primary feasibility outcome in Aim 2. There will be secondary feasibility outcomes (e.g., quantitative acceptability of the PAS intervention) in Aim 2.

### Engagement behavior in the PAS intervention

Engagement behavior at wave W, where W = W2, will be defined as the percentage of the number of post-introductory challenges completed in the PAS intervention at wave W: (*n*_post-introductory challenges completed W_/*n*_post-introductory challenges available_) × 100. The percentage will be used to assess the extent of usage in the PAS intervention. The lower bound threshold is < 40%. The upper bound threshold is ≥ 60%.

### Quantitative acceptability of the PAS intervention

Percentage of responses observed in “agree” or “strongly agree” to each of the 14 Likert-scale items designed to assess a subjective experience of the PAS intervention will be calculated at wave W, where W = W2. The lower bound threshold is < 60%. The upper bound threshold is ≥ 80%.

### Dichotomous acceptability of the PAS intervention

Percentage of responses observed in “yes” to the following item: “Would you recommend the online intervention to another person?” will be calculated at wave W, where W = W2. The lower bound threshold is < 60%. The upper bound threshold is ≥ 80%.

### Qualitative acceptability of the PAS intervention

Qualitative themes that emerge from a response to a qualitative item designed to assess the acceptability will be analyzed at wave W, where W = W2. The lower bound threshold is the presence of at least one potentially serious problem that is unable to be addressed in a future study. The upper bound threshold is the absence of the potentially serious problem.

### Quantitative acceptability of accelerometer-based assessment of PA

Percentage of responses observed in “agree” or “strongly agree” to each of the four Likert-scale items designed to assess the acceptability of accelerometer-based assessment of PA will be calculated at wave W, where W = W1 or W3. The lower bound threshold is < 60%. The upper bound threshold is ≥ 80%.

### Dichotomous acceptability of accelerometer-based assessment of PA

Percentage of responses observed in “yes” to the following item: “Would you be willing to wear the monitor again as part of a new research study?” will be calculated at wave W, where W = W1 or W3. The lower bound threshold is < 60%. The upper bound threshold is ≥ 80%.

### Qualitative acceptability of accelerometer-based assessment of PA

Qualitative themes that emerge from a response to qualitative items designed to assess the acceptability will be analyzed at wave W, where W = W1 or W3. The lower bound threshold is the presence of at least one potentially serious problem that is unable to be addressed in a future study. The upper bound threshold is the absence of the potentially serious problem.

### Aim 3

The Aim 3 is to provide a preliminary effect size estimate for each direct effect depicted in the conceptual model (see Fig. 1) for the PAS intervention. The preliminary effect size estimate for the direct effect of PAS to accelerometer-based assessment of PA will be the primary outcome in Aim 3. There will be secondary feasibility outcomes (e.g., PAS self-efficacy, self-efficacy PA) in Aim 3. The secondary feasibility outcomes in Aim 3 may provide additional information regarding the mechanism of the PAS intervention aimed at promoting PA. The preliminary effect sizes for each direct effect of being assigned to the PAS intervention at wave W, where W = W2 or W3 will be estimated. The lower bound threshold is < 0.00. The upper bound threshold is ≥ 0.20.

### Survey battery

Data on proposed demographic covariates of PA (e.g., gender, race, etc.) will be collected in the W1 survey battery [35]. Anthropometric data will be assessed by asking each participant their height and weight in the W1, W2, and W3 survey battery. The inclusion of the instruments (e.g., self-efficacy) is consistent with the previous FFW intervention research [46, 49].

### Self-efficacy to engage

After completion of the introductory challenges, PAS participants will be asked at W2 to respond to a newly developed scale designed to measure self-efficacy to engage. The 7-item scale is developed for this study based on literature pertaining to engagement with online behavioral interventions [50]. For example, the scale includes the following item: *How confident are you in your current ability to complete at least 24 post-introductory challenges in this online intervention within a 4-week time period?* Completing at least 24 post-introductory challenges within the next four weeks will be used to measure self-efficacy to engage based on substantive concerns (e.g., 2 h to complete at least 24 post-introductory challenges). A five-category rating scale structure was used for this item (i.e., 0 = no confidence to 4 = complete confidence), based on effective self-efficacy rating scale structures [51].

### PA self-efficacy

The PA self-efficacy will be measured from participants at W1, W2, and W3 with the PA self-efficacy scale [52]. The 48-item scale is a modified version of the exercise self-efficacy scale [53]. The PA self-efficacy scale was tailored for the PA context to assess the extent to which individuals believe that they have the ability to engage in a recommended amount of weekly PA for health. The scale measures weekly PA self-efficacy across the four PA domains (e.g., work-related PA). Each of the four domains has two unique stems that reference six increasing time periods.

### Self-efficacy to regulate PA

Self-efficacy to regulate PA will be measured from participants at W1, W2, and W3 with the self-efficacy to regulate PA scale [54]. The 13-item scale is a modified version of the barriers self-efficacy scale [55]. The self-efficacy to regulate PA scale was tailored for the PA context to assess the extent to which individuals believe that they have the ability to overcome possible barriers to engagement in a recommended amount of weekly PA for health. Specifically, the self-efficacy to regulate PA scale measures self-efficacy to regulate: (a) barriers to PA participation based on social considerations (e.g., one’s perception of another person’s evaluation of them like physical appearance) and (b) barriers to PA participation based on an internal subjective evaluation (e.g., an assessment of the ideality of the weather conditions).

### Acceptability of the PAS intervention

The acceptability of the PAS intervention will be assessed with PAS participants’ engagement data at W2. Consistent with the conceptualization of engagement with online interventions [50], both engagement behavior and subjective experience in the PAS intervention will be used to assess the acceptability of the intervention. Engagement behavior in the PAS intervention will be assessed at W2 by logging the number of post-introductory challenges completed by PAS participants from the PAS website. Subjective experience in the PAS intervention will be assessed by PAS participants at W2 with a modified questionnaire used in previous research [56]. The 16-item intervention acceptability questionnaire consists of a mix of 15 quantitative items with Likert-scale (i.e., 0 = strongly disagree to 4 = strongly agree) and one open-ended qualitative item.

### Acceptability of accelerometer-based assessment of PA

The acceptability of accelerometer-based assessment of PA will be assessed with a questionnaire used in previous research [46] at W1 and W3. The 11-item assessment acceptability questionnaire consists of a mix of six quantitative items with Likert-scale (e.g., 0 = strongly disagree to 4 = strongly agree) and five open-ended qualitative items.

### PA monitoring

Within 7 days after the W1 and W3 survey battery, participants will be asked to enter a code into the PAS intervention website in order to start wearing a PA monitor for the next consecutive 7 days. They will be instructed to wear a nylon belt around their waist with an accelerometer attached to it. Research staff will prepare the PA monitor package and meet each participant in the waiting room at the center to deliver the package by making an appointment. Along with a PA monitor attached to a belt, a cover letter, wear instructions, and a daily log sheet regarding wear time, will be included in the PA monitoring package. The cover letter will guide participants to use their login credentials on the PAS intervention website to enter a code to start wearing a PA monitor for the next consecutive 7 days. The wear instructions will provide full instructions with texts and pictures on how to complete the W1 and W3 PA monitoring. On the day after a 7-day interval, participants will receive a reminder by email and/or phone that prompts to use their login credentials on the website to complete instruments designed to measure the acceptability of accelerometer-based assessment of PA and self-reported PA.

Upon return of the monitor, data will be downloaded using a standard universal serial bus port. Within a few weeks of the return of the monitors, participants who provide usable data (to be described in the ActiGraph wGT3X-BT section) will be emailed a preliminary estimate of their wear time and average minutes per day of Moderate to Vigorous Physical Activity (MVPA) in relation to broad categories of recommended MVPA per week (i.e., 21.36 min/day or more). Participants who do not provide usable data will be informed that their average minutes per day of MVPA cannot be estimated due to insufficient data.

### ActiGraph wGT3X-BT

The ActiGraph wGT3X-BT (Pensacola, FL, USA) is a tri-axial research-grade accelerometer that will be used to measure PA objectively. ActiGraph devices have been used extensively as a reference device to measure free-living PA in adults [57]. Monitors will be initialized to collect raw acceleration data at 30 Hz using ActiLife software (v6.13.4). After download, data will be reintegrated to 60-s epochs. Non-wear time will be defined as ≥ 90 continuous minutes of zero counts, with allowance for 2 min of acceleration which are preceded and followed by at least 30 min of continuous zeros [58]. Usable data will be defined as follows: (a) a monitor is worn for at least 4 days including one weekend day with at least 10 h of valid wear time per day, (b) there is no evidence of monitor error, and (c) there is no issue from data compared to a log sheet. Unusable data will be treated as missing data. Average minutes per day of MVPA will be calculated based on established cut points (e.g., > 1952 counts per minute) [59].

### Self-reported PA

Self-reported PA will be measured with the long form of IPAQ [60]. This questionnaire purports to measure PA in the four domains according to the intensity, frequency, and duration of the PA performed in each domain during the previous week. Average minutes per day of MVPA will be calculated based on the IPAQ guidelines [61]. Self-reported PA will be included in the study because of the possibility of the inclusion in a definitive RCT with a large sample.

### Data analysis

Data analyses will include both quantitative and qualitative approaches. Quantitative analyses will be performed in M*plus* 8.0 under maximum-likelihood estimation with robust standard errors [62]. Missing data will be modeled under the assumption of missing at random [63]. Qualitative feedback will be summarized based on themes that emerge from the research team’s analysis. The threshold values for the traffic light system described in the feasibility outcome section will be used to evaluate results of specific indicators within each specific aim and with regard to the feasibility of a future definitive RCT. More specifically, descriptive statistics will be used to determine the feasibility of implementing the PAS intervention and the acceptability of implementing the PAS intervention. Intention-to-treat approach will be used to provide the preliminary effect size estimates [64]. The covariates, the outcome at W1, and group assignment will be specified as predictors in the approach.

## Discussion

We believe that the PAS intervention may have the potential to be effective in promoting PA in adults with obesity because of the three following reasons. First, the PAS intervention is based on self-efficacy theory to address the unique barriers to PA in adults with obesity [32–37]. Instead of typical PA advice, effective behavioral change techniques for the promotion of PA in adults with obesity are used in the PAS intervention based on previous research [23–25]. Second, a research-grade accelerometer is included in the implementation of the PAS intervention for the accurate evaluation of the program. This is important because recent findings reported less than high agreement in estimates of PA between accelerometer-based and self-report assessments [65]. Third, the implementation of the PAS intervention is in line with recent recommendations by the Community Preventive Services Task Force [66]. To be specific, implementing the PAS intervention includes activity monitors and promotes PA within a more broadly focused weight management program where there is access to a health care provider.

We are aware of at least three limitations for the feasibility study described in this protocol. The first limitation is that the recruitment of participants is to occur within a relatively controlled local context. Future research that investigates the feasibility and acceptability of implementing the PAS intervention for adults with obesity in a less controlled context (e.g., recruitment via a national health care panel recruitment company) may be worthwhile given the scientific utility of evaluating interventions in a variety of contexts [67, 68]. The second limitation is that each participant will be determined to be eligible for this study based on self-report. For some eligibility criteria (e.g., BMI), this limitation may be relatively minor due to a structural characteristic of the study design. For example, the eligibility criterion that BMI ≥ 25.00 kg/m^2^ should be truly met because enrollment in the weight management program provided by the center requires at least BMI ≥ 30.00 kg/m^2^. However, for some eligibility criteria (e.g., age), this limitation should be potentially more problematic due to the absence of a structural characteristic in the study design that can prevent the provision of false information. The third limitation deals with some uncertainty regarding the qualitative approach in aim 2. Although the qualitative approach (i.e., open-ended questions) in aim 2 to assess the acceptability of implementing the PAS intervention represents an extension of a more quantitatively focused questionnaire used in this study, it may still fail to collect some important information that a more rigorous qualitative approach (e.g., in-depth interviews) would provide.

Research suggests that adults with obesity should increase health-enhancing physical activity. We believe that an intervention for this important behavior change should be specifically developed based on evidence-based behavioral science and implemented with the use of technology and in collaboration with an external community partner. Results from the feasibility study described in this protocol are intended to inform the preparation of a future definitive RCT.

### Supplementary Information

**Supplementary Material 1.**

## Data Availability

Not applicable.

## Abbreviations

BMI: Body mass index
FFW: Fun for wellness
PA: Physical activity
PAS: Physical activity self-efficacy
SPIRIT: Standard protocol items: recommendations for interventional trials
UC: Usual care
USA: United states of America
W1: Wave 1
W2: Wave 2
W3: Wave 3
