# Supplementary material for: Physical activity self-efficacy online intervention for adults with obesity: protocol for a feasibility study
Source: Pilot Feasibility Stud. 2024 Feb 26;10:40. doi: 10.1186/s40814-024-01468-6 (PMC10895849; doi:10.1186/s40814-024-01468-6)
Supplement: Supplementary file 1 — Supplementary Material 1. [file 40814_2024_1468_MOESM1_ESM.docx]

Appendix A

**FFW Intervention**

Fun For Wellness (FFW) is a web-based behavioral intervention based on social cognitive theory [1,2] that promotes well-being and physical activity in adult populations by providing capability-enhancing opportunities. The capability-enhancing learning opportunities in the FFW intervention consist of 152 scenario-based and interactive challenges organized in the online environment by the acronym BET I CAN learning opportunities: Behavior (e.g., setting goals), Emotion (e.g., coping with negative emotions), Thought (e.g., imaging a new life story), Interaction (e.g., interacting with other people), Context (e.g., recognizing cues in the environment), Awareness (e.g., understanding themselves), and Next steps (e.g., making a plan). The learning opportunities in FFW target multiple domains of well-being (e.g., interpersonal, community, occupational, physical, psychological, economic domain) and physical activity in adult populations. The skills are taught by FFW to increase domain-specific self-efficacy, which, in turn, translate into an increase in the multi-dimensional well-being or physical activity. Previous research showed that the FFW intervention had the potential to be effective in promoting well-being and physical activity in adult populations [3-6].

**PAS Builds off FFW**

The Physical Activity Self-efficacy (PAS) intervention builds off the FFW intervention. Compared to FFW, the PAS intervention is more tailored for adults with obesity (i.e., at-risk subgroup of adults) and specifically designed to promote PA (i.e., not well-being) to achieve greater behavior change for their physical activity. Therefore, the capability-enhancing opportunities in the PAS intervention is different from the FFW intervention (see the precise reporting of the PAS intervention section). For example, the capability-enhancing learning opportunities in the PAS intervention focus on physical activity in adults with obesity (e.g., lectures on the importance of physical activity for adults with obesity, vignettes that are relevant to physical activity in adults with obesity). Because of the behavioral theory-based approach, the conceptual models and study designs between the PAS intervention and the FFW intervention are similar (e.g., self-efficacy construct targeted as a more proximal mediator of the concordant outcome).

**Precise Reporting of the PAS Intervention**

The PAS intervention is designed to provide four capability-enhancing opportunities, consistent with four sources of efficacy information: enactive mastery experiences, vicarious experiences, verbal persuasion, and physiological and/or emotional states in the PA domains. Each of the capability-enhancing opportunities in the PAS intervention is provided by one of the following intervention activities: play simple games and quizzes (i.e., enactive mastery experiences), watch vignettes (i.e., vicarious experiences), listen to short lectures (i.e., verbal persuasion), and engage in self-reflections and graded tasks for PA (i.e., physiological and/or emotional states). Also, the PAS intervention consists of effective behavioral change techniques for the population (e.g., action planning, teach to use prompts/cues, goal setting, etc.). The description of the components of the PAS interventions is provided in Table A.1.

Table A.1. Description of the Physical Activity Self-efficacy Intervention

| Components | Challenges | Behavioral Change Techniques^a^ | Contents |
| --- | --- | --- | --- |
| 1. introduction | introductory  challenge 1 | NA | overview of study |
|  | introductory  challenge 2 | NA | overview of intervention |
|  | introductory  challenge 3 | NA | review |
|  | introductory  challenge 4 | NA | assessment of self-efficacy to engage, based on previous research^7^ |
| 2. general education | post-introductory  challenge 1 | NA | lecture on definition of PA^8-10^ |
|  | post-introductory  challenge 2 | 5.1 information about health consequences,  5.2. salience of consequences | lecture on importance of PA^8-10^ |
|  | post-introductory  challenge 3 | 4.1 instruction on how to perform a behavior,  4.2 information about antecedents,  15.1 verbal persuasion about capability | lecture on tailored approaches to promote PA: part one^8-10^ |
|  | post-introductory  challenge 4 | 4.1 instruction on how to perform a behavior,  4.2 information about antecedents,  15.1 verbal persuasion about capability | lecture on tailored approaches to promote PA: part two^8-10^ |
|  | post-introductory  challenge 5 | 4.1 instruction on how to perform a behavior,  4.2 information about antecedents,  15.1 verbal persuasion about capability | lecture on tailored approaches to promote PA: part three^8-10^ |
|  | post-introductory  challenge 6 | NA | review |
| 3. self-efficacy source for  work-related PA | post-introductory  challenge 7 | 4.1 instruction on how to perform a behavior,  4.2 information about antecedents,  5.1 information about health consequences,  5.2. salience of consequences,  15.1 verbal persuasion about capability | lecture based on source of verbal persuasion for work-related PA^11^ |
|  | post-introductory  challenge 8 | 1.4 action planning,  8.3 habit formation,  8.7 graded tasks | source of physiological and/or emotional states for work-related PA^11^ |
|  | post-introductory  challenge 9 | 6.1 demonstration of the behavior | vignette based on source of vicarious experiences for work-related PA^11^ |
|  | post-introductory  challenge 10 | 7.1 prompts/cues,  15.2 mental rehearsal of successful performance,  15.3 focus on past success | source of enactive mastery experiences for work-related PA^11^ |
|  | post-introductory  challenge 11 | 1.1 goal setting (behavior),  1.3 goal setting (outcome),  3.1 social support (unspecified) | effective BCTs for adults with obesity^12-15^ |
| 4. self-efficacy source for  transport-related PA | post-introductory  challenge 12 | 4.1 instruction on how to perform a behavior,  4.2 information about antecedents,  5.1 information about health consequences,  5.2. salience of consequences,  15.1 verbal persuasion about capability | lecture based on source of verbal persuasion for transport-related PA^11^ |
|  | post-introductory  challenge 13 | 1.4 action planning,  8.3 habit formation,  8.7 graded tasks | source of physiological and/or emotional states for transport-related PA^11^ |
|  | post-introductory  challenge 14 | 6.1 demonstration of the behavior | vignette based on source of vicarious experiences for transport-related PA^11^ |
|  | post-introductory  challenge 15 | 7.1 prompts/cues,  15.2 mental rehearsal of successful performance,  15.3 focus on past success | source of enactive mastery experiences for transport-related PA^11^ |
|  | post-introductory  challenge 16 | 1.1 goal setting (behavior),  1.3 goal setting (outcome),  3.1 social support (unspecified) | effective BCTs for adults with obesity^12-15^ |
| 5. self-efficacy source for  domestic-related PA | post-introductory  challenge 17 | 4.1 instruction on how to perform a behavior,  4.2 information about antecedents,  5.1 information about health consequences,  5.2. salience of consequences,  15.1 verbal persuasion about capability | lecture based on source of verbal persuasion for domestic-related PA^11^ |
|  | post-introductory  challenge 18 | 1.4 action planning,  8.3 habit formation,  8.7 graded tasks | source of physiological and/or emotional states for domestic-related PA^11^ |
|  | post-introductory  challenge 19 | 6.1 demonstration of the behavior | vignette based on source of vicarious experiences for domestic-related PA^11^ |
|  | post-introductory  challenge 20 | 7.1 prompts/cues,  15.2 mental rehearsal of successful performance,  15.3 focus on past success | source of enactive mastery experiences for domestic-related PA^11^ |
|  | post-introductory  challenge 21 | 1.1 goal setting (behavior),  1.3 goal setting (outcome),  3.1 social support (unspecified) | effective BCTs for adults with obesity^12-15^ |
| 6. self-efficacy source for  leisure-related PA | post-introductory  challenge 22 | 4.1 instruction on how to perform a behavior,  4.2 information about antecedents,  5.1 information about health consequences,  5.2. salience of consequences,  15.1 verbal persuasion about capability | lecture based on source of verbal persuasion for leisure-related PA^11^ |
|  | post-introductory  challenge 23 | 1.4 action planning,  8.3 habit formation,  8.7 graded tasks | source of physiological and/or emotional states for leisure-related PA^11^ |
|  | post-introductory  challenge 24 | 6.1 demonstration of the behavior | vignette based on source of vicarious experiences for leisure-related PA^11^ |
|  | post-introductory  challenge 25 | 7.1 prompts/cues,  15.2 mental rehearsal of successful performance,  15.3 focus on past success | source of enactive mastery experiences for leisure-related PA^11^ |
|  | post-introductory  challenge 26 | 1.1 goal setting (behavior),  1.3 goal setting (outcome),  3.1 social support (unspecified) | effective BCTs for adults with obesity^12-15^ |

Note. PA = physical activity; BCTs = Behavioral Change Techniques; NA = Not Available.

^a^ Labels are consistent with the behavior change technique taxonomy (v1).^16^

**References**

1. Bandura A. Social foundations of thought and action: a social cognitive theory. Englewood Cliffs, New Jersey: Prentice-Hall; 1986
2. Bandura A. Health promotion from the perspective of social cognitive theory. Psychol Health. 1998;13:623-649.
3. Myers ND, Prilleltensky I, Prilleltensky O, et al. Efficacy of the fun for wellness online intervention to promote multidimensional well-being: a randomized controlled trial. Prev Sci. 2017;18:984-994. doi:10.1007/s11121-017-0779-z
4. Myers ND, McMahon A, Prilleltensky I, et al. Effectiveness of the fun for wellness web-based behavioral intervention to promote physical activity in adults with obesity (or overweight): randomized controlled trial. JMIR Form Res. 2020;4(2):e15919. doi:10.2196/15919
5. Myers ND, Prilleltensky I, McMahon A, et al. Effectiveness of the fun for wellness online behavioral intervention to promote subjective well-being in adults with obesity: a randomized controlled trial. J Happiness Stud. 2021;22:1905-1923. doi:10.1007/s10902-020-00301-0
6. Lee S, McMahon A, Prilleltensky I, et al. Effectiveness of the fun for wellness online behavioral intervention to promote well-being actions in adults with obesity or overweight: A randomized controlled trial. J Sport Exerc Psychol. 2020;43(1):83-96. doi:10.1123/jsep.2020-0049
7. Jo B. Model misspecification sensitivity analysis in estimating causal effects of interventions with non-compliance. Stat Med. 2002;21:3161-3181. doi:10.1002/sim.1267
8. Physical Activity Guidelines Advisory Committee. 2018 physical activity guidelines advisory committee scientific report. U.S. Department of Health and Human Services; 2018. Accessed July 30, 2023. https://health.gov/sites/default/files/2019-09/PAG_Advisory_Committee_Report.pdf
9. World Health Organization: Global recommendations on physical activity for health. Accessed July 30, 2023. http://apps.who.int/iris/bitstream/10665/44399/1/9789241599979_eng
10. American Heart Association editorial staff. American heart association recommendations for physical activity in adults and kids. American Heart Association; 2018. Accessed July 30, 2023. https://www.heart.org/en/healthy-living/fitness/fitness-basics/aha-recs-for-physical-activity-in-adults
11. Bandura A. Self-efficacy: toward a unifying theory of behavioral change. Psychol Rev. 1977;84:191-215. doi:10.1037/0033-295X.84.2.191
12. Samdal GB, Eide GE, Barth T, et al. Effective behaviour change techniques for physical activity and healthy eating in overweight and obese adults; systematic review and meta-regression analyses. Int J Behav Nutr Phys Act. 2017;14:1-4. doi:10.1186/s12966-017-0494-y
13. Olander EK, Fletcher H, Williams S, et al. What are the most effective techniques in changing obese individuals’ physical activity self-efficacy and behaviour: a systematic review and meta-analysis. Int J Behav Nutr Phys Act. 2013;10(1):1-5. doi:10.1186/1479-5868-10-29
14. Carraça E, Encantado J, Battista F, et al. Effective behavior change techniques to promote physical activity in adults with overweight or obesity: a systematic review and meta‐analysis. Obes Rev. 2021;22:e13258. doi:10.1111/obr.13258
15. Williams SL, French DP. What are the most effective intervention techniques for changing physical activity self-efficacy and physical activity behaviour-and are they the same? Health Educ Res. 2011;26:308-322. doi:10.1093/her/cyr005
16. Michie S, Richardson M, Johnston M, et al. The behavior change technique taxonomy (v1) of 93 hierarchically clustered techniques: building an international consensus for the reporting of behavior change interventions. Ann Behav Med. 2013;46(1):81-95. doi:10.1007/s12160-013-9486-6

Appendix B

Table B.1. A Populated SPIRIT Checklist

SPIRIT 2013 Checklist: Recommended items to address in a clinical trial protocol and related documents*

| Section/item | Item No | Description | Addressed on page number |
| --- | --- | --- | --- |
| **Administrative information** | | |  |
| Title | 1 | Descriptive title identifying the study design, population, interventions, and, if applicable, trial acronym | 1 |
| Trial registration | 2a | Trial identifier and registry name. If not yet registered, name of intended registry | 3,6 |
|  | 2b | All items from the World Health Organization Trial Registration Data Set | not applicable |
| Protocol version | 3 | Date and version identifier | 3,6 |
| Funding | 4 | Sources and types of financial, material, and other support | 3,20 |
| Roles and responsibilities | 5a | Names, affiliations, and roles of protocol contributors | 1,20 |
|  | 5b | Name and contact information for the trial sponsor | 3,20 |
|  | 5c | Role of study sponsor and funders, if any, in study design; collection, management, analysis, and interpretation of data; writing of the report; and the decision to submit the report for publication, including whether they will have ultimate authority over any of these activities | not applicable |
|  | 5d | Composition, roles, and responsibilities of the coordinating centre, steering committee, endpoint adjudication committee, data management team, and other individuals or groups overseeing the trial, if applicable (see Item 21a for data monitoring committee) | not applicable |
| Introduction |  |  |  |
| Background and rationale | 6a | Description of research question and justification for undertaking the trial, including summary of relevant studies (published and unpublished) examining benefits and harms for each intervention | 4-6 |
|  | 6b | Explanation for choice of comparators | 6-8 |
| Objectives | 7 | Specific objectives or hypotheses | 5-6 |
| Trial design | 8 | Description of trial design including type of trial (eg, parallel group, crossover, factorial, single group), allocation ratio, and framework (eg, superiority, equivalence, noninferiority, exploratory) | 6 |
| Methods: Participants, interventions, and outcomes | | |  |
| Study setting | 9 | Description of study settings (eg, community clinic, academic hospital) and list of countries where data will be collected. Reference to where list of study sites can be obtained | 6-7 |
| Eligibility criteria | 10 | Inclusion and exclusion criteria for participants. If applicable, eligibility criteria for study centres and individuals who will perform the interventions (eg, surgeons, psychotherapists) | 7 |
| Interventions | 11a | Interventions for each group with sufficient detail to allow replication, including how and when they will be administered | 8-9, appendix |
|  | 11b | Criteria for discontinuing or modifying allocated interventions for a given trial participant (eg, drug dose change in response to harms, participant request, or improving/worsening disease) | not applicable |
|  | 11c | Strategies to improve adherence to intervention protocols, and any procedures for monitoring adherence (eg, drug tablet return, laboratory tests) | 8-12 |
|  | 11d | Relevant concomitant care and interventions that are permitted or prohibited during the trial | 8-9 |
| Outcomes | 12 | Primary, secondary, and other outcomes, including the specific measurement variable (eg, systolic blood pressure), analysis metric (eg, change from baseline, final value, time to event), method of aggregation (eg, median, proportion), and time point for each outcome. Explanation of the clinical relevance of chosen efficacy and harm outcomes is strongly recommended | 9-12 |
| Participant timeline | 13 | Time schedule of enrolment, interventions (including any run-ins and washouts), assessments, and visits for participants. A schematic diagram is highly recommended (see Figure) | 8-9,Figure 2 |
| Sample size | 14 | Estimated number of participants needed to achieve study objectives and how it was determined, including clinical and statistical assumptions supporting any sample size calculations | 7 |
| Recruitment | 15 | Strategies for achieving adequate participant enrolment to reach target sample size | 6-8 |
| **Methods: Assignment of interventions (for controlled trials)** | | |  |
| Allocation: |  |  |  |
| Sequence generation | 16a | Method of generating the allocation sequence (eg, computer-generated random numbers), and list of any factors for stratification. To reduce predictability of a random sequence, details of any planned restriction (eg, blocking) should be provided in a separate document that is unavailable to those who enrol participants or assign interventions | 8 |
| Allocation concealment mechanism | 16b | Mechanism of implementing the allocation sequence (eg, central telephone; sequentially numbered, opaque, sealed envelopes), describing any steps to conceal the sequence until interventions are assigned | 8 |
| Implementation | 16c | Who will generate the allocation sequence, who will enrol participants, and who will assign participants to interventions | 8 |
| Blinding (masking) | 17a | Who will be blinded after assignment to interventions (eg, trial participants, care providers, outcome assessors, data analysts), and how | 8 |
|  | 17b | If blinded, circumstances under which unblinding is permissible, and procedure for revealing a participant’s allocated intervention during the trial | 8 |
| **Methods: Data collection, management, and analysis** | | |  |
| Data collection methods | 18a | Plans for assessment and collection of outcome, baseline, and other trial data, including any related processes to promote data quality (eg, duplicate measurements, training of assessors) and a description of study instruments (eg, questionnaires, laboratory tests) along with their reliability and validity, if known. Reference to where data collection forms can be found, if not in the protocol | 12-16 |
|  | 18b | Plans to promote participant retention and complete follow-up, including list of any outcome data to be collected for participants who discontinue or deviate from intervention protocols | 12-16 |
| Data management | 19 | Plans for data entry, coding, security, and storage, including any related processes to promote data quality (eg, double data entry; range checks for data values). Reference to where details of data management procedures can be found, if not in the protocol | 12-16,appendix |
| Statistical methods | 20a | Statistical methods for analysing primary and secondary outcomes. Reference to where other details of the statistical analysis plan can be found, if not in the protocol | 16 |
|  | 20b | Methods for any additional analyses (eg, subgroup and adjusted analyses) | 16 |
|  | 20c | Definition of analysis population relating to protocol non-adherence (eg, as randomised analysis), and any statistical methods to handle missing data (eg, multiple imputation) | 16 |
| **Methods: Monitoring** | | |  |
| Data monitoring | 21a | Composition of data monitoring committee (DMC); summary of its role and reporting structure; statement of whether it is independent from the sponsor and competing interests; and reference to where further details about its charter can be found, if not in the protocol. Alternatively, an explanation of why a DMC is not needed | not applicable |
|  | 21b | Description of any interim analyses and stopping guidelines, including who will have access to these interim results and make the final decision to terminate the trial | not applicable |
| Harms | 22 | Plans for collecting, assessing, reporting, and managing solicited and spontaneously reported adverse events and other unintended effects of trial interventions or trial conduct | not applicable |
| Auditing | 23 | Frequency and procedures for auditing trial conduct, if any, and whether the process will be independent from investigators and the sponsor | not applicable |
| Ethics and dissemination | | |  |
| Research ethics approval | 24 | Plans for seeking research ethics committee/institutional review board (REC/IRB) approval | 6 |
| Protocol amendments | 25 | Plans for communicating important protocol modifications (eg, changes to eligibility criteria, outcomes, analyses) to relevant parties (eg, investigators, REC/IRBs, trial participants, trial registries, journals, regulators) | 3,6 |
| Consent or assent | 26a | Who will obtain informed consent or assent from potential trial participants or authorised surrogates, and how (see Item 32) | 6-7 |
|  | 26b | Additional consent provisions for collection and use of participant data and biological specimens in ancillary studies, if applicable | not applicable |
| Confidentiality | 27 | How personal information about potential and enrolled participants will be collected, shared, and maintained in order to protect confidentiality before, during, and after the trial | appendix |
| Declaration of interests | 28 | Financial and other competing interests for principal investigators for the overall trial and each study site | 20 |
| Access to data | 29 | Statement of who will have access to the final trial dataset, and disclosure of contractual agreements that limit such access for investigators | not applicable |
| Ancillary and post-trial care | 30 | Provisions, if any, for ancillary and post-trial care, and for compensation to those who suffer harm from trial participation | not applicable |
| Dissemination policy | 31a | Plans for investigators and sponsor to communicate trial results to participants, healthcare professionals, the public, and other relevant groups (eg, via publication, reporting in results databases, or other data sharing arrangements), including any publication restrictions | not applicable |
|  | 31b | Authorship eligibility guidelines and any intended use of professional writers | not applicable |
|  | 31c | Plans, if any, for granting public access to the full protocol, participant-level dataset, and statistical code | not applicable |
| Appendices |  |  |  |
| Informed consent materials | 32 | Model consent form and other related documentation given to participants and authorised surrogates | not applicable |
| Biological specimens | 33 | Plans for collection, laboratory evaluation, and storage of biological specimens for genetic or molecular analysis in the current trial and for future use in ancillary studies, if applicable | not applicable |

*It is strongly recommended that this checklist be read in conjunction with the SPIRIT 2013 Explanation & Elaboration for important clarification on the items. Amendments to the protocol should be tracked and dated. The SPIRIT checklist is copyrighted by the SPIRIT Group under the Creative Commons “[Attribution-NonCommercial-NoDerivs 3.0 Unported](http://www.creativecommons.org/licenses/by-nc-nd/3.0/)” license.

Appendix C

**Confidentiality**

The feasibility study will have procedures in place to limit privacy risks. Participants will be asked to provide some personally identifiable information (e.g., phone number) during enrollment in the study. This personally identifiable information and all other data collected online (e.g., online survey battery) will be collected via a secure website maintained by authorized research staff employed by Lehigh University. The research staff will share all data collected via the secure website with authorized research staff employed by Binghamton University. Data obtained from this research will be stored in such a way that human participants cannot be identified because all account holders will be assigned a coded identification number in the stored data. The list connecting each participant with an identification number will be kept protected, encrypted, private, and only accessible to authorized research staff at Lehigh University and Binghamton University.

Appendix D

**Eligibility**

There are six eligibility criteria for participation in this study. Values for each of the eligibility criteria will be based on self-report by the potential participant. Potential participants who do not meet one or more of the eligibility criteria will be informed that they are ineligible for the study and then will be taken to the exit page from the PAS website.

The first and the second eligibility criteria focus on physical characteristics of the potential participant. The first criterion is being between 18 and 64 years old, consistent with evidence-based age groupings for global recommendations on physical activity for health [1,2]. The second criterion is a Body Mass Index (BMI) ≥ 25.00 kg/m^2^, consistent with many physical activity interventions in adults with obesity [3-5]. A justification for this criterion is the need to promote physical activity in a BMI-based population in which few individuals may meet public health guidelines for physical activity [6,7].

The other eligibility criteria focus on the interaction of the potential participant and the online intervention study. The third criterion is the ability to access the online intervention. This criterion will be assessed by asking them to confirm that they will have access to a technological device (e.g., computer, smart phone) that can access the online intervention via a web browser. The fourth criterion is the absence of simultaneous enrollment in another intervention to promote PA (not counting the weight management program). The fifth criterion is a willingness to comply with instructions for physical activity monitoring. This criterion will be assessed by asking them if they are willing to wear a nylon belt around their waist with an accelerometer attached to it for two 7-day intervals and complete a daily log sheet regarding wear time during each of the two 7-day intervals in the study. A justification for this criterion is based on previous studies where a similar question was asked to potential participants at screening [8,9]. Those who indicate a willingness to comply with instructions for physical activity monitoring will be subsequently asked to provide information about their non-dominant hand. The sixth criterion is a willingness to respond to study-related contacts (e.g., email, text message, phone call). A justification for this criterion is that participants will be asked to complete the study-related tasks in a specifically structured schedule.

**References**

1. Physical Activity Guidelines Advisory Committee. 2018 physical activity guidelines advisory committee scientific report. U.S. Department of Health and Human Services; 2018. Accessed July 30, 2023. https://health.gov/sites/default/files/2019-09/PAG_Advisory_Committee_Report.pdf
2. World Health Organization: global recommendations on physical activity for health; 2010. Accessed 30 July, 2023. http://apps.who.int/iris/bitstream/10665/44399/1/9789241599979_eng
3. Lee S, Patel P, Myers ND, et al. A systematic review of eHealth interventions to promote physical activity in adults with obesity or overweight. Behav Med. 2023; 49(3):213-230. doi:10.1080/08964289.2022.2065239
4. Gourlan MJ, Trouilloud DO, Sarrazin PG. Interventions promoting physical activity among obese populations: a meta-analysis considering global effect, long-term maintenance, physical activity indicators and dose characteristics. Obes Rev. 2011;12:e633-e645. doi:10.1111/j.1467-789X.2011.00874.x
5. de Vries HJ, Kooiman TJM, van Ittersum MW, et al. Do activity monitors increase physical activity in adults with overweight or obesity? A systematic review and meta-analysis. Obesity. 2016;24:2076-2091. doi:10.1002/oby.21619
6. Tran L, Tran P, Tran L. A cross-sectional examination of sociodemographic factors associated with meeting physical activity recommendations in overweight and obese US adults. Obes Res Clin Pract. 2020;14(1):91-98. doi:10.1016/j.orcp.2020.01.002
7. Tudor-Locke C, Brashear MM, Johnson WD, et al. Accelerometer profiles of physical activity and inactivity in normal weight, overweight, and obese U.S. men and women. Int J Behav Nutr Phys Act. 2010;7(1):60. doi:10.1186/1479-5868-7-60
8. Howard VJ, Rhodes D, Mosher A, et al. Obtaining accelerometer data in a national cohort of black and white adults. Med Sci Sports Exec. 2015;47:1531-1537. doi:10.1249/MSS.0000000000000549
9. Myers ND, Lee S, Bateman AG, et al. Accelerometer-based assessment of physical activity within the Fun For Wellness online behavioral intervention: protocol for a feasibility study. Pilot Feasibility Stud. 2019;5(1):1-8. doi:10.1186/s40814-019-0455-0
